# Modified Scleral Needling Technique to Achieve Scleral Wound Closure in Microincision Vitrectomy Surgery

**DOI:** 10.7759/cureus.84700

**Published:** 2025-05-23

**Authors:** Ichihiko Takeuchi, Hiroyuki Nakashizuka, Chiho Shoda, Masanori Iwasaki, Koji Tanaka, Ryusaburo Mori

**Affiliations:** 1 Ophthalmology, Nihon University School of Medicine, Tokyo, JPN; 2 Ophthalmology, Nihon University Hospital, Tokyo, JPN

**Keywords:** intraocular pressure, scleral needling technique, sclerotomy, small incision vitrectomy surgery, vitrectomy, wound closure techniques

## Abstract

Purpose: The study aimed to evaluate the effectiveness of the modified scleral needling (SN) technique for unclosed scleral wounds in 25-gauge vitrectomy.

Methods: This retrospective study examined 50 scleral wounds that failed to self-close at the end of vitrectomy surgery performed by the same surgeon using a three-port 25-gauge vitrectomy system (Constellation® Vision System, Alcon Laboratories, Inc., Fort Worth, TX, USA) at Nihon University Hospital between February and August 2023. In all cases, scleral wounds were created by oblique puncture, and sufficient peripheral vitrectomy was performed. After the removal of the cannula, wound closure was assessed under air perfusion. The modified SN technique was implemented if the scleral wounds, excluding the infusion port, did not self-close. If self-closure was not achieved after three attempts of the modified SN technique, the scleral wound was sutured.

Results: The modified SN technique closed 98% (49 of 50) of the leaking scleral wounds, and the average number of punctures was 1.36. Cochran's Q test revealed a statistically significant difference in the rate at which air leaks were sealed across the four stages (Q = 117.97; p = 2.11 × 10⁻²⁵). Subsequent pairwise comparisons using McNemar's test showed a significant improvement between the zero (no treatment) and first applications (p = 0.0005), as well as between the first and second applications (p = 0.0156). However, there was no statistically significant difference between the second and third applications (p = 0.125).

Conclusion: The modified SN technique is safe and effective for promoting scleral wound closure in microincision vitrectomy surgery.

## Introduction

In microincision vitrectomy surgery, incomplete scleral wound closure is a risk factor for endophthalmitis and postoperative hypotony [1]; thus, ensuring complete wound closure is essential for preventing postoperative complications. When the self-closure of sclerotomies fails, the following methods are effective for closing the sclerotomies: injecting water into the scleral wound [2], suturing with suture thread [3], injecting viscoelastic material under the conjunctiva near the wound [4], applying tissue adhesive to the wound [5], and cauterizing the conjunctiva at the wound [6]. Felfeli et al. devised the scleral needling (SN) technique, which promotes wound closure by inserting a 30-gauge (G) needle into scleral wounds, and reported that the number of cases requiring sutures after the introduction of the SN technique decreased from 29% to 1% with no adverse events observed [7]. We conducted a follow-up study using a method similar to the SN technique previously reported by Felfeli et al. Although the basic concept was based on their approach, our method differed in that we used a 30-G needle without a syringe attachment, allowing intraocular air to escape during the procedure. In this study, we report the usefulness of this modified technique for achieving sclerotomy closure.

## Materials and methods

Study design

We conducted a retrospective study examining 50 scleral wounds that failed to self-close at the end of microincision vitrectomy surgeries, which were performed by the same surgeon using a three-port 25-G vitrectomy system (Constellation® Vision System, Alcon Laboratories, Inc., Fort Worth, TX, USA) at Nihon University Hospital in Tokyo, Japan, between February and August 2023. Patients with scleral wounds at the site of the infusion port were excluded from the study. There were 34 scleral ports in 24 male patients with a mean age of 63.11 ± 11.6 years and 16 scleral ports in 11 female patients with a mean age of 61.0 ± 6.7 years. Twenty-eight patients underwent simultaneous cataract surgery, and seven patients underwent vitrectomy alone. There were no cases of lens-sparing vitrectomy. Vitrectomy was performed in 14 patients with epiretinal membranes, 12 with macular holes, three with silicone oil removal, three with rhegmatogenous retinal detachment, and three with proliferative diabetic retinopathy. For scleral wounds that failed to self-close, the modified SN technique was performed to achieve closure. This study adhered to the tenets of the Declaration of Helsinki and was approved by the Institutional Review Board of Nihon University Hospital (approval number: 20240104).

Surgical technique

**Figure 1:**
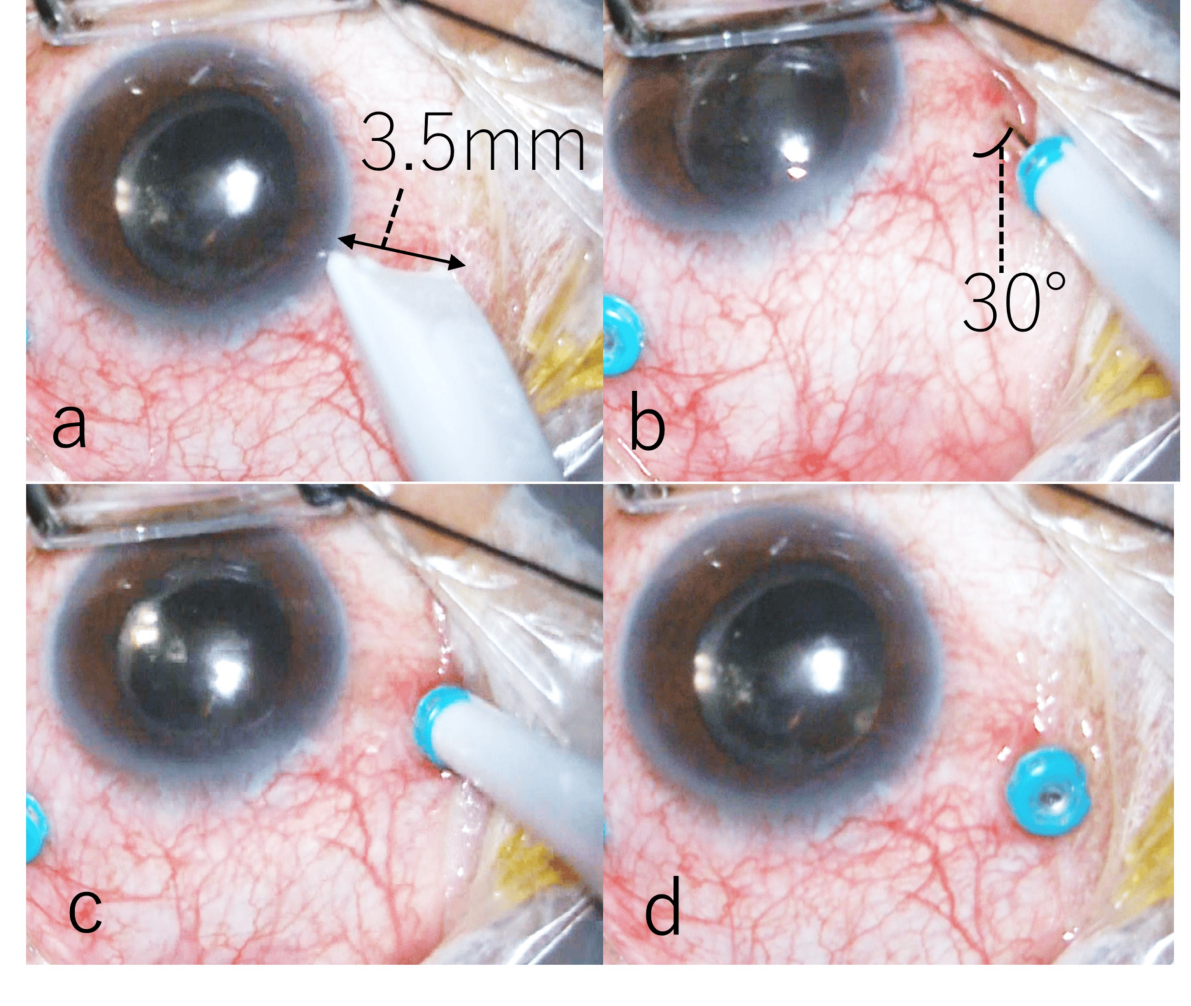
Creation of sclerotomies A bi-bevel trocar was inserted at a 30-degree oblique angle at a site 3.5 mm from and parallel to the corneal limbus (a), creating a tunneled sclerotomy (b). Then, the trocar was advanced vertically toward the center of the vitreous body (c) and withdrawn, leaving the cannula in place (d).

**Figure 2:**
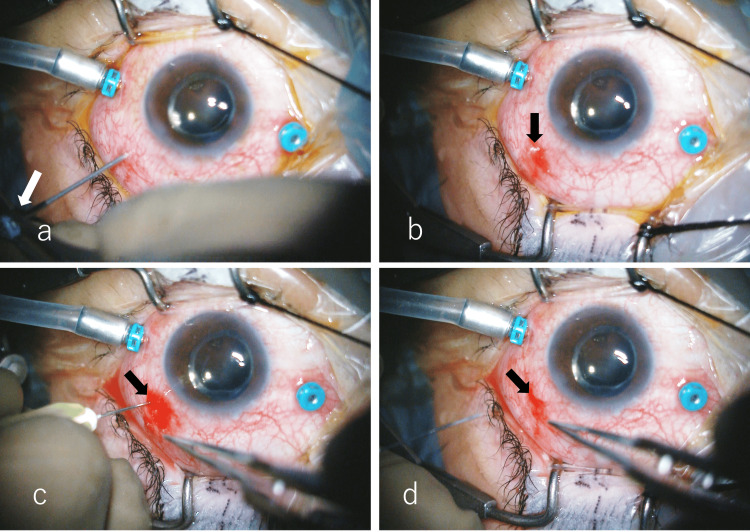
Removal of the cannula The cannula (white arrow) was removed before the vitreous cutter to prevent vitreous herniation at the sclerotomy site (a). The scleral wound was compressed with forceps one to three times over the conjunctiva to close the scleral valves of the sclerotomies. Water was dripped onto the wounds, and in case the presence of air bubbles was noted (black arrow) from the wounds (b), modified SN was performed on the scleral tunnel (black arrow) with a 30-G needle without syringe attachment. Briefly, the 30-G needle was inserted between the outer and inner lips of the sclerotomy, slightly inferior to the original entry site (i.e., toward the patient's feet) (c). The maneuver was performed over approximately 3-4 seconds to allow controlled air escape through the needle. After removing the needle, the scleral lips (black arrow) were gently compressed again with forceps, applying minimal blunt pressure to avoid tissue damage (d). SN: scleral needling

In phakic eye cases, cataract surgery was followed by vitrectomy. At a site 3.5 mm from and parallel to the corneal limbus (Figure 1a), a bi-bevel trocar was inserted at a 30-degree oblique angle, creating a tunneled sclerotomy (Figure 1b). The trocar was then advanced vertically toward the center of the vitreous body (Figure 1c) and withdrawn, leaving the cannula in place [8] (Figure 1d). During surgery, the vitrectomy cutter was operated at 10,000 cuts per minute with a maximum vacuum pressure of 650 mmHg. All patients underwent sufficient peripheral vitrectomy with scleral compression, including the scleral wound site, under direct microscopic observation. At the end of the vitrectomy, approximately 30% of the vitreous cavity was fluid-air exchanged while maintaining an air perfusion pressure of 35 mmHg. A vitreous cutter was inserted into the cannula of the remaining two ports except the infusion port. The vitreous cutter was then driven into the cannula to prevent vitreous herniation at the sclerotomy site. At this time, the cut rate was set at 10,000 cuts per minute, and the vacuum pressure was reduced to approximately 150 mmHg. The cannula was removed before the vitreous cutter (Figure 2a). The scleral wound was compressed with forceps one to three times over the conjunctiva to close the scleral valves of the sclerotomies. Water was dripped onto the wounds, and the presence or absence of air bubbles from the wounds was used to determine whether the wounds were closed (Figure 2b). A wound that did not close after three scleral compressions was defined as a failure of self-wound closure. In these cases, the SN technique was performed on the scleral tunnel using a simplified approach with a 30-G needle alone, without syringe attachment (NIPRO, Inc., Bridgewater, NJ, USA). Briefly, the 30-G needle was inserted between the outer and inner lips of the sclerotomy, slightly inferior to the original entry site (i.e., toward the patient's feet) (Figure 2c). The maneuver was performed over approximately 3-4 seconds to allow controlled air escape through the needle. After removing the needle, the scleral lips were gently compressed again with forceps, applying minimal blunt pressure to avoid tissue damage (Figure 2d). If wound closure was not achieved even after three attempts using the SN method, the scleral wound was sutured with a 7-0 Vicryl thread (MANI, Inc., Tochigi, Japan) (Video 1).

**Video 1:** Modified SN technique for the unclosed scleral wound in microincision vitrectomy surgery At the end of the vitrectomy, approximately 30% of the vitreous cavity was fluid-air exchanged while maintaining an air perfusion pressure of 35 mmHg. A vitreous cutter was inserted into the cannula of the remaining two ports except the infusion port. The vitreous cutter was then driven into the cannula to prevent vitreous herniation at the sclerotomy site. The cannula was removed before the vitreous cutter. The scleral wound was compressed with forceps one to three times over the conjunctiva to close the scleral valves of the sclerotomies. Water was dripped onto the wounds, and the presence or absence of air bubbles from the wounds was used to determine whether the wounds were closed. A wound that did not close after three scleral compressions was defined as a failure of self-wound closure. In these cases, the modified SN technique was performed on the scleral tunnel with a 30-G needle without syringe attachment (NIPRO, Inc., Bridgewater, NJ, USA). If wound closure was not achieved even after three attempts using the SN method, the scleral wound was sutured with a 7-0 Vicryl thread (MANI, Inc., Tochigi, Japan). SN: scleral needling

Statistical analysis

Statistical analyses were performed to evaluate the effectiveness of the adhesive in sealing air leaks. The data were analyzed using Cochran's Q test to determine whether the proportion of sealed wounds changed significantly across different application times (zero, one, two, and three applications). If Cochran's Q test showed statistical significance (p < 0.05), McNemar's test was conducted for pairwise comparisons between each application step (zero vs. one, one vs. two, and two vs. three applications) to identify specific differences. A p-value of <0.05 was considered statistically significant. Statistical analyses were performed using Python's SciPy and Statsmodels libraries. All statistical analyses were conducted using Python version 3.11.8 (Python Software Foundation, Wilmington, DE, USA). Cochran's Q test and McNemar's test were performed using the SciPy (version 1.9.3) and Statsmodels (version 0.13.5) libraries.

## Results

The modified SN method closed 49 of the 50 non-closed wounds, resulting in a closure rate of 98%. Suture closure was employed in one patient who underwent simultaneous cataract surgery and vitrectomy for the epiretinal membrane (axial length: 24.1 mm). The average number of punctures required to close the wound was 1.36 ± 0.72 (one puncture: 38/50 (76%); two punctures: 7/50 (14%), three punctures: 4/50 (8%); suture closure: 1/50 (2%)) (Table 1).　

**Table 1:** Outcomes of wound closure using the modified SN technique (n = 50) This table summarizes the number and percentage of eyes in which sclerotomy closure was achieved using the SN technique. Closure was obtained after one, two, or three punctures in 49 out of 50 cases. One case required suture placement. The mean number of punctures required for closure was 1.36 ± 0.72, with an overall closure rate of 98%. SN: scleral needling

Closure method	Number of eyes	Percentage
Closed after 1 puncture	38	76%
Closed after 2 punctures	7	14%
Closed after 3 punctures	4	8%
Required suture	1	2%
Total	50	100%
Mean number of punctures: 1.36 ± 0.72		
Overall closure rate: 98% (49/50)		

After the modified SN technique, 14 eyes were treated with sulfur hexafluoride (SF6) or perfluoropropane (C3F8) gas tamponade. However, there were no cases of air leakage from the eye, even during air-gas exchange. The mean intraocular pressure measured with a noncontact meter on the day after surgery was 10.9 ± 6.4 mmHg. Four patients with an intraocular pressure of 5 mmHg or less underwent the Seidel test using fluorescein strips to detect the leakage of aqueous humor, and all four patients were negative for Seidel's sign (Table 2). Postoperative vitreous hemorrhage was observed in one eye, but the findings at reoperation showed no association with the SN technique.

**Table 2:** Postoperative observations following the modified SN technique This table presents postoperative findings in eyes treated with the modified SN technique. Fourteen eyes received gas tamponade with SF6 or C3F8, but no cases of air leakage were observed, including during air-gas exchange. The mean intraocular pressure on postoperative day 1 was 10.9 ± 6.4 mmHg. Four eyes with hypotony (≤5 mmHg) underwent the Seidel testing, and all were negative for aqueous leakage. SN: scleral needling; SF6: sulfur hexafluoride; C3F8: perfluoropropane

Parameter	Result
Gas tamponade (SF6 or C3F8)	14 eyes
Intraocular pressure (day 1)	10.9 ± 6.4 mmHg
Seidel test (eyes with ≤5 mmHg)	4 eyes tested, all negative
Air leakage after SN technique including during air-gas exchange	None observed

Cochran's Q test revealed a statistically significant difference in the rate at which air leaks were sealed across the four stages (Q = 117.97; p = 2.11 × 10⁻²⁵). Subsequent pairwise comparisons using McNemar's test showed a significant improvement between the zero (no treatment) and first applications (p = 0.0005), as well as between the first and second applications (p = 0.0156). However, there was no statistically significant difference between the second and third applications (p = 0.125) (Table 3).

**Table 3:** McNemar's test results McNemar's test was used to compare the rate at which air leaks were sealed after each sequential puncture. The comparison includes a baseline "no puncture" condition (i.e., no treatment) followed by one, two, or three punctures. P-values less than 0.05 were considered statistically significant. "Yes" in the "significant (p < 0.05)" column indicates a significant difference between the compared puncture stages.

Comparison	P-value	Significant (p < 0.05)
0 vs. 1 puncture	0.000488	Yes
1 vs. 2 punctures	0.015625	Yes
2 vs. 3 punctures	0.125	No

## Discussion

The SN technique has been proposed as an alternative method for scleral wound closure, offering a less invasive option compared to traditional suturing. In this study, we used a modified version of the SN technique, which, while based on the original concept from Felfeli et al. [7], involves using a 30-G needle without a syringe attachment, simplifying the procedure.

**Figure 3:**
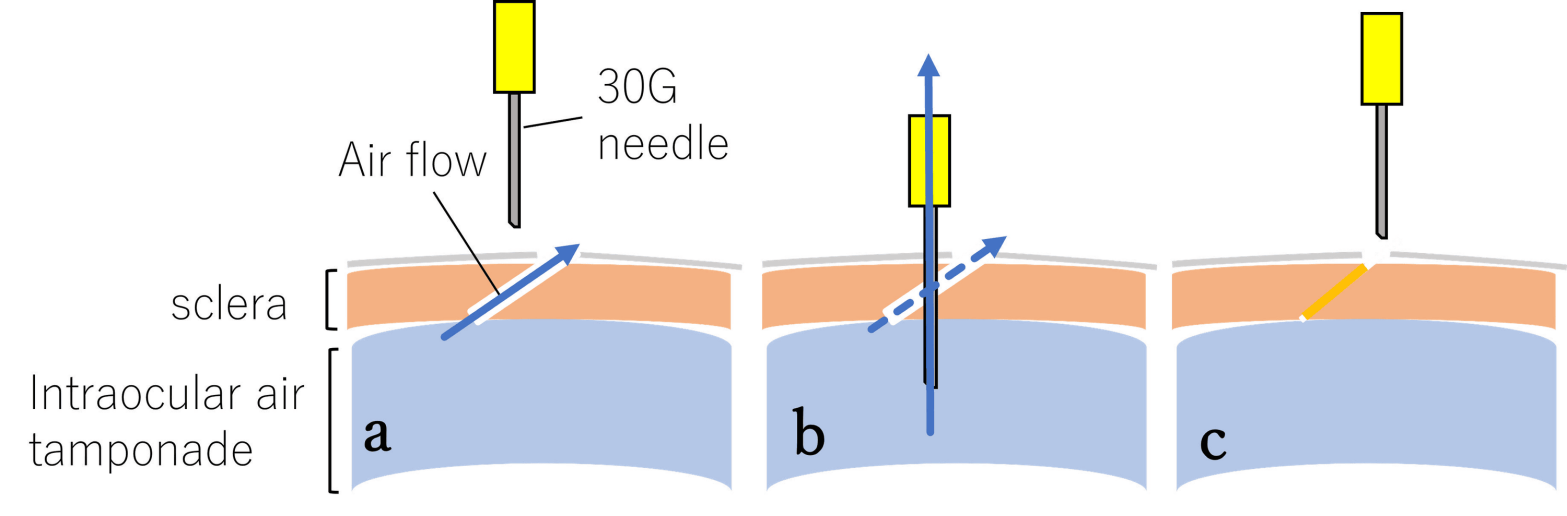
Proposed wound closure mechanism 1 after the modified SN technique A 30-G needle insertion creates an air outflow pathway other than the sclerotomy site, reducing relative airflow volume through the sclerotomy (a, b) and potentially accelerating the closure of the sclerotomy wound (c). However, the exact mechanism remains speculative, and further studies are needed to confirm its role. This figure is an original work created by the authors and has not been published elsewhere. SN: scleral needling

**Figure 4:**
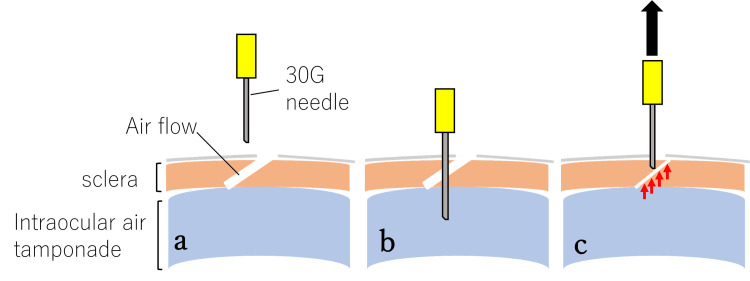
Proposed wound closure mechanism 2 after SN techniques The 30-G needle insertion and removal might also mechanically raise the internal valve of the scleral tunnel (red arrows) at the sclerotomy site (a, b, c). However, this mechanism remains hypothetical, and further studies are required to confirm its role in wound closure. This figure is an original work created by the authors and has not been published elsewhere. SN: scleral needling

The closure rate after the modified SN method in this study was high at 98% (49/50 sites). However, the exact mechanism of wound closure using these techniques remains unclear. Felfeli et al. [7] speculated that the vitreous may become drawn into the interior of the needle and flow into the wound sites during the removal of the needle. In a previous report, an endoscope illumination experiment using enucleated porcine eyes confirmed that vitreous herniation occurred at the scleral wound during intravitreal injection, even using a 30-G needle in porcine non-vitrectomized eyes [9]. In this study, peripheral vitrectomy was performed while applying scleral compression around the sclerotomy sites, and the cutter was driven inside the cannula during cannula removal to prevent vitreous incarceration as much as possible. However, it might be impossible to remove the vitreous body completely, and there is a possibility that the residual vitreous body may have caused vitreous herniation. Nevertheless, even in three cases of silicone oil removal, in which the residual vitreous near the wound was considered less and solid, wound closure was achieved using this technique. This finding suggests that there may be a mechanism other than vitreous herniation. As another possible mechanism, we considered the possibility that the 30-G needle insertion creates an air outflow pathway other than the sclerotomy, reducing airflow volume through the sclerotomy relatively (Figure 3a, 3b) and accelerating the closure of the sclerotomy wound (Figure 3c). This mechanism does not apply at all to the wound closure mechanism in Felfeli et al.'s method, where air outflow was restricted using a syringe. However, we have experienced cases in which air leakage from the sclerotomy site stopped immediately upon insertion of a 30-G needle over the scleral flap, before the needle was withdrawn. Furthermore, a previous report indicated that the sclerotomy was closed by pushing up the internal valve with a trocar [10]. The 30-G needle insertion and removal might also mechanically pull up the internal valve of the scleral tunnel at the sclerotomy site (Figure 4a, 4b, 4c). We hypothesized that a combination of these mechanisms might promote wound closure. However, the proposed mechanisms (airflow redirection and mechanical repositioning of scleral lips by needle manipulation) are speculative and currently lack sufficient objective evidence. While needle placement and angle of insertion are critical factors, we explicitly state that these mechanisms remain hypothetical and acknowledge that other factors, such as transient intraocular pressure reduction, could significantly contribute to wound closure.

When sclerotomies do not close, wound closure with absorbable sutures is usually performed. However, sutures can cause allergic keratoconjunctivitis and may cause long-term foreign body sensations. In addition, the SN techniques are more straightforward and inexpensive than sutures. We believe that the SN techniques can be attempted before suturing. In this study, postoperative vitreous hemorrhage was observed in one eye; however, reoperation findings showed no association with the SN technique. Nevertheless, vitreous hemorrhage can occur with the needling technique. Since no significant difference was found between the second and third punctures, it seems reasonable to limit the procedure to two attempts. If the sclerotomy does not close after two attempts, suturing should be performed without hesitation. In this study, the infusion cannula site was excluded from the evaluation to ensure an accurate assessment of the remaining two sclerotomy sites using the needling technique. This decision was made not only to allow the precise evaluation of leakage at those sites but also because hypotony can occur after infusion cannula removal, in which case suturing should be prioritized over the needling technique. In fact, in this study, suturing was performed at two infusion sclerotomy sites. Especially for less experienced surgeons, it is considered safer to proceed with suturing rather than persisting with repeated needling.

This study has several limitations. First, all surgeries were performed by a single experienced surgeon. Consequently, the observed closure rate may not be generalizable to broader clinical settings, particularly when performed by less experienced surgeons or under prolonged surgical conditions that place stress on the sclerotomy site. Second, as this was a retrospective study, selection bias may have influenced the study population, and the relatively small sample size limits the generalizability of our findings. Third, not all potential postoperative complications, such as subconjunctival hemorrhage, were documented, despite a previous report by Felfeli et al. [7] indicating a higher incidence of such events with the SN techniques. We explicitly acknowledge this limitation and recommend that future studies specifically examine the occurrence of subconjunctival hemorrhage during SN procedures. Fourth, this study did not include postoperative wound assessment using anterior segment optical coherence tomography (AS-OCT), which could have provided a more objective and detailed evaluation of scleral wound closure. We explicitly acknowledge this limitation, and future prospective studies should incorporate intraoperative AS-OCT imaging to confirm and objectively illustrate the proposed wound closure mechanism. This would significantly enhance the precision of surgical outcome assessments and provide a more reliable basis for evaluating the effectiveness of the technique. Fifth, the exact mechanism by which repeated punctures promote sclerotomy closure remains unclear. Observational studies using ex vivo porcine eyes combined with intraocular endoscopy may help elucidate the wound dynamics and structural changes involved in this phenomenon. Moreover, operative time and the frequency of instrument insertion and removal through the sclerotomy site were not documented in this study. These factors could potentially influence the mechanical stress applied to the sclerotomy, which might affect the likelihood of requiring repeated SN attempts for closure. We explicitly acknowledge the absence of these details in our study. Future studies should consider documenting these factors, as they may provide valuable insights into the wound closure dynamics and the reproducibility of the technique. Further prospective studies involving larger patient cohorts are warranted to address these limitations and to validate the broader applicability of the SN techniques.

## Conclusions

We applied the modified SN technique to 50 scleral wounds and found it to be a simple, safe, and effective method for achieving high wound closure rates in cases of incomplete sclerotomy closure. This technique offers a less invasive alternative to suturing, potentially reducing postoperative discomfort and suture-related complications such as foreign body sensation and allergic reactions. However, to further validate its efficacy and long-term safety, multicenter, prospective comparative studies with larger sample sizes and extended follow-up periods are necessary. Furthermore, additional research is warranted to elucidate the underlying mechanisms by which the modified SN technique facilitates sclerotomy closure. Given its advantages in simplicity, cost-effectiveness, and reduced patient burden, the modified SN technique may be considered as a first-line approach before resorting to suture placement for unsealed sclerotomies.
